# Notes and News

**Published:** 1901-07-13

**Authors:** 


### NOTES AND NEWS.

THE Metropolitan Hospital Sunday Fund collection already exceeds £46,000.

CASES of plague at the Cape and Port Elizabeth are now dwindling down to the smallest proportions.

THE Festival Dinner of the Royal Free Hospital will be held on Monday, July 15th, at the Hôtel Cecil.

PROFESSOR RUDOLF VIRCHOW has been created a Knight of the Prussian order "Pour la Mérite" for sciences and art.

THE medical profession are giving a complimentary dinner to Surgeon-General Jameson, C.B., at the Hôtel Cecil on Wednesday, July 24th.

THE man who threw a piece of iron some time past at the German Emperor has been declared by medical experts to be an epileptic, and irresponsible. He is to be placed in an asylum for observation.

THE late Mr. Thomas Elsey, of Hilperton's Marsh, near Trowbridge, has bequeathed £1,000 each to the Bristol Royal Infirmary, The Children's Hospital on St. Michael's Hill, Bristol, and the Queen Victoria Convalescent Home, Bristol, and £500 to the Trowbridge Cottage Hospital. The Bristol Royal Infirmary is also residuary legatee to the extent of about £15,000.

THE foundation-stone of a new cottage hospital for Southwold has been laid by the Countess of Stradbroke. Hitherto old buildings adapted have been used for the purpose. The new building is to contain two large and two small wards, operating room, and the usual offices, together with an out-patients' department. All the money necessary to complete the hospital has not yet been collected.

AT the recent election to the Council of the Royal College of Surgeons the ballot resulted as follows:—Mr. Arthur William Mayo Robson, 332 votes; Mr. William Watson Cheyne, 302; Mr. Richard Clement Lucas, 259; Mr. John Hammond Morgan, 236; Mr. Charles William Mansell Moullin, 235; Mr. Henry Hugh Clutton, 145; Mr. John Bland Sutton, 193; Mr. Jordan Lloyd, 93. The President declared Mr. Mayo Robson and Mr. Watson Cheyne duly re-elected, and Mr. R. C. Lucas duly elected.

THE Bishop of London has consented to become a vice-president of the Metropolitan Hospital Saturday Fund. Since January last the workshop collections have produced £7,000.

THE Asylums Committee of the London County Council have decided to put an end to the arrangement under which for the past six years the medical staff at Claybury Asylum has included two lady doctors. It is stated that the arrangement has not proved altogether satisfactory.

THE United States Government have decided not to permit anyone suffering from tuberculosis to land at any of its ports. The rule first applied to emigrants, but it now includes first-class passengers. There are some favourite health resorts for consumptives in the United States, now alien patients will, we conclude, be debarred from frequenting them.

THE Committee of the National Refuges for Homeless and Destitute Children desire us to state that there are vacancies on board the *Arethusa* and *Chichester* training ships for poor boys of good character who wish to go to sea. The vacancies arise because so many lads have recently been sent into the Royal Navy and Mercantile Marine. Mission workers, school teachers, and all others interested in the welfare of these boys, may be thankful to hear of such openings for aspiring young sailors of good character. The ages should be between 14 and 16, and applications may be sent addressed to the Secretary, 164 Shaftesbury Avenue, W.C., at which address he interviews candidates every morning at 11 o'clock. All suitable cases are admitted at once without votes.

THE principal events at the British Congress on Tuberculosis which commences on July 22nd, will be the General Meeting, when the Duke of Cambridge will open the Congress, on July 23rd Professor Koch's address, on July 24th the address by Professor Brouardel, of Paris, and that of Professor McFadyean on July 25th. Meetings of sections will take place daily between 9.30 and 1.30, and after 4.30 daily the programme is entirely devoted to social functions. The Congress closes on July 27th with a garden party given by the Duke and Duchess of Northumberland at Syon. Previous to the opening of the opening of the Congress the reception room at the Queen's Hall will be open on July 20th and 21st, when names will be registered and tickets distributed from 10 A.M. to 1 P.M.

THE Quarterly General Court of Governors of St. George's Hospital held yesterday was to be made special, among other things, to consider, and, if approved, to enact, the following as a law of the hospital: "That previous to the adoption in a medical case of a method of treatment regarded as involving serious risk, a consultation should, as far as is consistent with the interests of the patient, be held between two or more members of the medical staff." In a little hospital, where one man may possibly become a dominant influence, it may be well to have such a rule as this, but in a great hospital like St. George's, with a medical school attached and with students, the most severe of critics, all round, the propriety of loosening responsibility by demanding a formal and probably perfunctory consultation is greatly to be doubted.

